# Development of Novel Predictive Scores for Obstetrical Risk Stratification in Adolescent Pregnancies: A Retrospective Study

**DOI:** 10.3390/jcm15010139

**Published:** 2025-12-24

**Authors:** Abdul Jabar Khudor, Marius Alexandru Moga, Oana Gabriela Dimienescu, Andrada Camelia Nicolau, Cristian Andrei Arvatescu, Mircea Daniel Hogea

**Affiliations:** 1 Medicine PhD School, Transilvania University of Brasov, 500036 Brașov, Romania; 2 Faculty of Medicine, Transilvania University of Brasov, 500036 Brașov, Romania

**Keywords:** adolescent pregnancy, obstetrical risk, predictive scores, CRUI, ADOLESRISK, cervical ripening, pregnancy complications, risk stratification

## Abstract

**Background**: Adolescent pregnancies represent a significant global health challenge, with increased risks of maternal and neonatal complications. Traditional obstetrical risk assessment tools have limited applicability in this population due to unique physiological and anatomical characteristics. This study aimed to develop and validate novel predictive scores specifically designed for obstetrical risk stratification in adolescent pregnancies. **Methods**: A retrospective study was conducted over seven years (2018–2024) in Brasov County, Romania, including 1322 adolescent pregnancies (ages 12–16 years). Two novel predictive scores were developed: the Cervical Ripening Ultrasound Index (CRUI) for predicting successful vaginal delivery and labor induction, and the ADOLESRISK score for comprehensive obstetrical risk stratification. Statistical analysis included logistic regression, ROC curve analysis, and validation testing using SPSS 26.0 and R Studio version 4.3.2. **Results**: The CRUI score demonstrated superior predictive performance (AUC = 0.87, 95% CI: 0.84–0.90) compared to traditional Bishop score (AUC = 0.62, 95% CI: 0.58–0.66) for successful labor induction in adolescents. The ADOLESRISK score achieved 84% sensitivity and 76% specificity for predicting major obstetrical complications, significantly outperforming conventional risk assessment tools. Key risk factors incorporated included maternal age, educational level, nutritional status, and specific ultrasound parameters. Internal validation using train–test split methodology (70–30%) confirmed robust performance in the independent validation cohort (n = 397), with maintained discriminative ability (CRUI: AUC = 0.85, 95% CI: 0.80–0.90; ADOLESRISK: AUC = 0.82, 95% CI: 0.77–0.87) across different demographic subgroups. **Conclusions**: The CRUI and ADOLESRISK scores represent significant advances in adolescent obstetrical care, providing clinicians with tools for personalized risk assessment and management. Implementation of these scores could potentially reduce maternal complications by 25–30% and improve neonatal outcomes by 20–25%, representing a major contribution to adolescent reproductive health globally.

## 1. Introduction

Adolescent pregnancy remains a critical global health issue. According to World Health Organization data, approximately 21 million girls aged 15–19 years become pregnant annually in developing regions, with about 12 million proceeding to birth. This represents approximately 13% of all births occurring among adolescents under 18 years of age. Additionally, at least 777,000 girls under 15 years give birth annually worldwide, facing particularly elevated health risks due to extreme physiological immaturity [1,2]. Despite declining trends in many developed countries, adolescent pregnancies continue to pose significant challenges due to increased risks of maternal and neonatal complications, including anemia, preterm birth, preeclampsia, low birth weight, and increased perinatal mortality [3,4,5,6,7].

The physiological and anatomical characteristics of adolescents create unique obstetrical challenges that distinguish this population from adult pregnancies. Incomplete pelvic development, ongoing somatic growth, and psychosocial immaturity contribute to increased complications and require specialized management approaches [8,9,10,11,12]. Furthermore, adolescent pregnancies are often associated with socioeconomic disadvantages, including lower educational attainment, inadequate prenatal care, and limited access to healthcare resources [13,14,15,16,17].

Current obstetrical risk assessment tools, including traditional scoring systems such as the bishop score for cervical assessment and various maternal risk stratification models, were primarily developed and validated in adult populations [18,19,20,21,22,23]. These tools demonstrate limited accuracy and clinical utility when applied to adolescent pregnancies, largely due to the unique biological and social characteristics of this population [21,22,24,25,26]. The inadequacy of existing predictive models necessitates the development of age-specific tools that can accurately assess obstetrical risks and guide clinical decision-making in adolescent pregnancies [27,28,29]. Romania presents a particularly relevant context for studying adolescent pregnancies, with one of the highest adolescent birth rates in the European Union [30,31,32,33,34]. According to Eurostat data from 2023, Romania accounts for nearly 25% of all mothers under 15 years in the EU, with significant disparities based on educational level and geographic location [31,35,36,37]. This demographic diversity provides an ideal setting for developing and validating predictive tools that can be applied across different population subgroups [33,34,38].

The primary objectives of this study were to: develop and validate the Cervical Ripening Ultrasound Index (CRUI) for predicting successful vaginal delivery and labor induction outcomes in adolescents, to create and validate the ADOLESRISK score for comprehensive obstetrical risk stratification, to compare the performance of these novel tools with traditional risk assessment methods and to provide evidence-based recommendations for implementing personalized care protocols in adolescent obstetrical management.

The COVID-19 pandemic profoundly disrupted healthcare systems worldwide, with significant implications for maternal and perinatal care during 2020–2021. The implementation of lockdown measures, travel restrictions, and social distancing policies fundamentally altered healthcare behaviors among pregnant women and the delivery of obstetric services. Multiple studies have documented a substantial decrease in antenatal clinic attendance, with reductions ranging from 29% to 45% in various settings, even as delivery rates remained stable [39,40]. Women cited fear of contracting SARS-CoV-2 infection in healthcare facilities and transportation difficulties during lockdowns as primary barriers to accessing prenatal care [41]. This reduction in routine antenatal visits was accompanied by decreased attendance for essential screening procedures, including ultrasound examinations, antenatal infection screening, and prenatal genetic testing [42]. Furthermore, evidence suggests a significant decline in unscheduled care visits to obstetric emergency departments, with a increase in the proportion of pregnant women requiring hospitalization among those who did present for urgent care, potentially indicating more advanced disease at presentation due to delayed care-seeking [43]. The reorganization of maternity services during the pandemic led to rapid implementation of telemedicine and virtual antenatal care protocols, particularly in high-income countries [40,44]. Restrictions on birth companions during labor and delivery, shortened postpartum hospital stays, and limitations on family visitation further contributed to increased maternal psychological distress [45]. The pandemic’s impact extended beyond direct healthcare access, significantly affecting maternal mental health and well-being. Studies consistently demonstrated elevated rates of depression and anxiety among pregnant women during the COVID-19 pandemic compared to pre-pandemic cohorts [44,46]. Risk factors for compromised maternal [44,47] mental health included previous psychiatric disorders, being in the third trimester of pregnancy, social isolation, lack of social support, financial difficulties, and fear of COVID-19 infection affecting the mother or fetus.

These pandemic-related disruptions in healthcare access and increased psychological burden may have contributed to adverse maternal and perinatal outcomes observed during this period, including increased rates of stillbirth, maternal mortality, and maternal intensive care unit admissions in certain populations [42,48,49]. The effect of delayed presentation to care resulted in more advanced complications at diagnosis, including anemia, post-term pregnancy, and pregnancy-induced hypertension [41]. Although pregnant women [50] with COVID-19 infection generally did not experience more severe disease symptoms than non-pregnant individuals, those with symptomatic infection, particularly in the third trimester, faced increased risks of intensive care admission, mechanical ventilation, preterm delivery, and cesarean section. The consequences of the COVID-19 pandemic on obstetric care delivery and maternal health behaviors underscore the importance of maintaining essential reproductive health services during public health emergencies, ensuring equitable access to care regardless of socioeconomic status, and providing adequate psychological support to pregnant women facing unprecedented stressors.

## 2. Materials and Methods

### 2.1. Study Design

This retrospective single-center study was conducted over seven consecutive years (January 2018 to December 2024) at the main tertiary obstetrical center serving Brasov County, Romania (Clinical Hospital of Obstetrics and Gynecology “Dr.I.A.Sbarcea”). Written informed consent was obtained from all participants or their legal guardians for minors under 16 years of age at the time of admission.

### 2.2. Participants and Inclusion Criteria

The study included all adolescent pregnancies meeting the following criteria: maternal age between 12 and 16 years at delivery; singleton pregnancies; delivery at the study institution during the study period; complete medical records available for analysis; and informed consent for data utilization. Exclusion criteria included: multiple pregnancies (twin or higher-order gestations, n = 18 excluded), major fetal anomalies detected on prenatal ultrasound (n = 7 excluded), and incomplete medical records preventing adequate risk assessment (n = 23 excluded). The final study cohort comprised 1322 adolescent pregnancies, representing a consecutive, exhaustive sample of all cases meeting inclusion criteria during the study period.

The final study cohort comprised 1322 adolescent pregnancies, representing a consecutive, exhaustive sample of all cases meeting inclusion criteria during the study period.

### 2.3. Data Collection Procedures

Comprehensive data collection included demographic characteristics (age, educational level, residence, socioeconomic status), medical history, prenatal care utilization (when available), anthropometric measurements, laboratory parameters, ultrasound findings, labor and delivery details and neonatal outcomes. Educational attainment was assessed as a binary variable based on completion of lower secondary education (gymnasium, grades V–VIII in the Romanian education system, typically completed by age 14–15). This classification reflects not chronological impossibility of completing education, but rather school engagement and educational trajectory prior to pregnancy. For adolescents aged 15–16 years at delivery (84.1% of our cohort), “secondary education completed” indicates successful completion of the 8th grade (gymnasium) before pregnancy occurrence, representing age-appropriate educational progression. For younger adolescents (12–14 years, 15.9% of cohort), these variable captures whether they were enrolled in and progressing through school at grade-appropriate levels versus early school dropout or non-enrollment. Data was obtained from maternal self-report at hospital admission. The educational system context is relevant: in rural Romania (82.8% of our cohort), school dropout rates are substantially higher than urban areas, and early pregnancy itself is both a cause and consequence of educational disengagement, creating bidirectional relationships that our cross-sectional measurement cannot fully disentangle.

Specific attention was paid to parameters relevant to adolescent physiology, including nutritional status, growth velocity, and psychosocial factors.

Ultrasound examinations were performed by certified maternal-fetal medicine specialists using standardized protocols on Voluson E8 system (GE Healthcare, Pfaffing, Austria). Cervical assessment included measurement of cervical length, posterior cervical angle, cervical volume, and echogenicity parameters. Advanced biometric parameters were recorded, including estimated fetal weight, amniotic fluid volume, and Doppler studies when indicated.

Adequacy of prenatal care was classified as:

▪ Adequate: ≥4 prenatal visits with first visit initiated before 20 weeks gestation, including baseline laboratory evaluation (complete blood count, blood type, infectious disease screening) and at least two ultrasound examinations (first trimester dating scan and second-trimester anomaly scan).
▪ Inadequate: <4 prenatal visits OR first visit after 20 weeks gestation, regardless of total visit number.
▪ Absent: No documented prenatal visits before delivery.

This classification enabled systematic assessment of healthcare utilization patterns and their association with pregnancy outcomes.

### 2.4. Outcome Definitions

Primary outcomes were defined as binary variables to enable predictive score development and validation.

For CRUI Score evaluation:

▪ Favorable outcome: Successful vaginal delivery within 24 h of labor induction initiation or spontaneous labor leading to vaginal delivery without complications requiring operative intervention.
▪ Unfavorable outcome: Failed induction requiring cesarean section or prolonged labor exceeding 24 h from induction initiation or operative vaginal delivery (vacuum) for failure to progress.

For ADOLESRISK Score evaluation:

▪ Favorable outcome: Uncomplicated pregnancy and delivery without major complications as defined below.
▪ Unfavorable outcome: Occurrence of one or more major complications, defined as:
  - Severe preeclampsia: Systolic blood pressure ≥ 160 mmHg or diastolic blood pressure ≥ 110 mmHg on two occasions at least 4 h apart, plus proteinuria ≥ 5 g/24 h (or protein/creatinine ratio ≥ 0.3) or adverse conditions (thrombocytopenia, renal insufficiency, liver dysfunction, pulmonary edema, cerebral symptoms).
  - Eclampsia: New-onset grand mal seizures in a woman with preeclampsia, without other identifiable cause.
  - Preterm birth: Spontaneous or medically indicated delivery before 37 completed weeks of gestation.
  - Cesarean delivery: Performed for maternal indication (cephalopelvic disproportion, failed induction) or fetal indication (non-reassuring fetal status, malpresentation, cord prolapse).
  - Postpartum hemorrhage: Estimated blood loss > 500 mL after vaginal delivery or > 1000 mL after cesarean delivery, or any blood loss sufficient to cause hemodynamic instability or require blood transfusion.
  - Fetal growth restriction: Estimated fetal weight < 10th percentile for gestational age with abnormal umbilical artery Doppler findings.
  - Neonatal intensive care unit (NICU) admission: Neonatal admission to intensive care unit for >24 h for respiratory distress, hypoglycemia, sepsis evaluation, or other medical indication.
  - Perinatal death: Stillbirth (fetal death after 24 completed weeks of gestation) or early neonatal death (death within 7 days of birth).

### 2.5. Score Development Methodology

*CRUI Score Development*: The Cervical Ripening Ultrasound Index (CRUI) was developed using a composite approach incorporating multiple ultrasound parameters: cervical length (CL), cervical echogenicity score (CES), posterior cervical angle (PCA) and presence of funneling (PF). A weighted scoring system was created based on logistic regression coefficients, with each parameter contributing proportionally to its predictive value for successful vaginal delivery.

ADOLESRISK Score Development: The ADOLESRISK score was developed through multivariate analysis of factors significantly associated with major obstetrical complications, including preeclampsia, preterm birth, cesarean delivery, and adverse neonatal outcomes. The scoring algorithm incorporated maternal age, body mass index, educational level, prenatal care adequacy, hemoglobin levels, and specific ultrasound parameters.

The ADOLESRISK score calculation formula is:

ADOLESRISK Score = (Age points) + (BMI points) + (Education points) + (Prenatal care points) + (Hemoglobin points) + (Gestational age points) + (EFW points)

Point allocation based on multivariate logistic regression coefficients are explained in Table 1:

Cutoff values were determined through receiver operating characteristic (ROC) curve analysis using Youden index optimization (J = sensitivity + specificity − 1) to balance sensitivity and specificity for predicting major obstetrical complications. The cutoff of ≥12 points maximized this index, providing optimal discrimination between favorable and unfavorable outcomes.

To assess the robustness, reproducibility, and potential generalizability of the developed predictive scores, we performed internal validation using a random train–test split approach, a widely accepted method for evaluating predictive model performance when external validation cohorts from independent institutions are unavailable. The complete study cohort (N = 1322) was randomly divided into two distinct subsets:

▪ Training set: 70% of total cohort (n = 925), used exclusively for score development, including variable selection, logistic regression modeling, coefficient estimation, weighting determination, and optimal cutoff value identification.
▪ Validation set: 30% of total cohort (n = 397), reserved exclusively for independent performance evaluation without any involvement in model development processes.

Both training and validation datasets were compared for demographic characteristics (age, residence, education), clinical parameters (BMI, hemoglobin, prenatal care adequacy), and outcome distributions using Chi-square tests for categorical variables and independent samples *t*-tests for continuous variables. No statistically significant differences were detected between training and validation cohorts for any baseline characteristic (all *p* > 0.05), confirming successful randomization and balanced distribution.

The derived scoring algorithms (including specific coefficients, point allocations, and cutoff values) were then applied without any modification or recalibration to the validation dataset. This approach simulates real-world application where scores developed in one population are applied to new patients.

The following metrics were calculated separately for both training and validation cohorts: area under the receiver operating characteristic curve (AUC) with 95% confidence intervals using DeLong method, sensitivity, specificity, positive predictive value (PPV), and negative predictive value (NPV) at optimal cutoff, calibration assessed using Hosmer–Lemeshow goodness-of-fit test (χ^2^ statistic with *p* > 0.05 indicating adequate calibration), comparison of AUC between training and validation cohorts using DeLong test for correlated ROC curves.

This internal validation approach provides preliminary evidence of model performance stability and indicates potential generalizability within similar populations. However, it is important to note that both training and validation cohorts derive from the same institution and time period, sharing common characteristics including healthcare system structure, provider practices, and patient population demographics. Therefore, while internal validation demonstrates absence of severe overfitting, external validation in independent cohorts from different geographic locations, healthcare settings, and demographic contexts remain essential before widespread clinical implementation can be recommended.

### 2.6. Statistical Analysis

Statistical analysis was performed using SPSS version 26.0 (IBM Corp., Armonk, NY, USA), Excel 2019 (Microsoft Corp., Redmond, WA, USA), and R Studio version 4.3.2. Descriptive statistics included means, standard deviations, frequencies, and percentages. Comparative analyses utilized Chi-square tests, Student’s *t*-test, ANOVA, and Mann–Whitney U test as appropriate. Logistic regression models were developed for score creation, and receiver operating characteristic (ROC) curve analysis was performed to assess predictive performance. Statistical significance was set at *p* < 0.05. Performance metrics included sensitivity, specificity, positive and negative predictive values, and area under the curve (AUC) with 95% confidence intervals.

The potential clinical utility and impact of implementing the developed scores in routine adolescent obstetrical care were evaluated through multiple complementary approaches: decision curve analysis (DCA) to assess net benefit across different probability thresholds, comparing the scores against “treat all patients as high-risk” and “treat no patients as high-risk” strategies, with net benefit calculated as [True Positives/n] − [False Positives/n] × [pt/(1 − pt)], where pt represents probability threshold; number needed to screen (NNS) calculation to determine how many adolescents would require risk assessment to identify one case of major complication, calculated as 1/(sensitivity × prevalence); potential intervention impact estimation, modeling scenarios where high-risk patients (score ≥ 15) receive intensive monitoring protocol (weekly prenatal visits, biweekly ultrasound assessments, serial growth scans, antenatal corticosteroids when indicated, planned early-term delivery) and moderate-risk patients (score 8–14) receive enhanced standard care (biweekly visits, monthly ultrasound), with complication reduction estimates based on published intervention studies in adolescent populations showing 20–30% reduction with intensive monitoring; resource allocation modeling comparing predicted resource utilization patterns (total ultrasound examinations, specialist consultations, NICU bed-days) between risk-stratified care protocols versus standard uniform care approach, calculating potential cost implications. Statistical comparisons utilized Chi-square tests for categorical outcomes and Z-tests for comparing proportions between groups, with *p* < 0.05 considered statistically significant.

### 2.7. AI Statement

The authors declare that GenAI was used in the creation of this manuscript. The authors confirm full responsibility for the use of Gen AI in the preparation of this manuscript. GenAI was applied exclusively for language refinement, including grammar, style, clarity, and conciseness, as well as for harmonizing figure legends, structuring sections, and formatting references. All scientific ideas, model development, data analyses, and interpretations were performed independently by the authors. Software versions: Grammarly Premium (Version 14.1162.0) was used for grammar and style refinement, for language editing, harmonizing figure legends, and structuring manuscript sections was used by Microsoft Copilot (Microsoft 365). Microsoft Word for Microsoft 365 (Version 2412, Build 18324.20140) was used for manuscript preparation. Formatting and reference management was performed using Mendeley Desktop (Version 1.19.8).

## 3. Results

### 3.1. Demographic Characteristics

The study cohort of 1322 adolescent pregnancies had a mean maternal age of 15.3 ± 0.8 years, with 84.1% of deliveries occurring in the 15–16-year age group. Geographic distribution revealed 82.8% rural residence, reflecting the demographic characteristics of the region. Educational attainment was notably low, with only 27.5% having completed secondary education. Prenatal care utilization was suboptimal, with 34.2% receiving inadequate prenatal care according to standard guidelines. Nutritional status assessment revealed significant deficiencies, with 45% of participants presenting with anemia (hemoglobin < 11 g/dL) and 28% with low body mass index for gestational age. This data is summarized in Table 2.

Table 3 presents the distribution of pregnancy and neonatal outcomes in the study population. Vaginal delivery occurred in 73.8% of cases, while cesarean delivery was performed in 26.2%. The composite outcome of any major complication was present in 29.4% of pregnancies. Mean birth weight was 2987 ± 512 g, with low birth weight (<2500 g) in 18.4% of neonates. NICU admission was required in 21.0% of cases, and total perinatal mortality rate was 0.9%.

To confirm successful randomization and absence of systematic differences that could bias validation results, we performed comprehensive statistical comparisons between training and validation cohorts across all baseline demographic, clinical, and outcome variables (Table 4). Chi-square tests were employed for categorical variables (age groups, residence, education, binary clinical indicators), while independent samples *t*-tests were used for continuous normally distributed variables (age, BMI, hemoglobin, gestational age). No statistically significant differences were detected for any characteristic (all *p* > 0.05), confirming that randomization successfully created balanced cohorts suitable for unbiased validation assessment. This balance verification is critical for internal validation, as systematic differences between training and validation cohorts would compromise the validity of performance comparisons and potentially overestimate or underestimate true model generalizability.

No statistically significant differences were detected between training and validation cohorts for any baseline characteristic (all *p* > 0.05), confirming successful randomization and balanced distribution.

**Figure 1:**
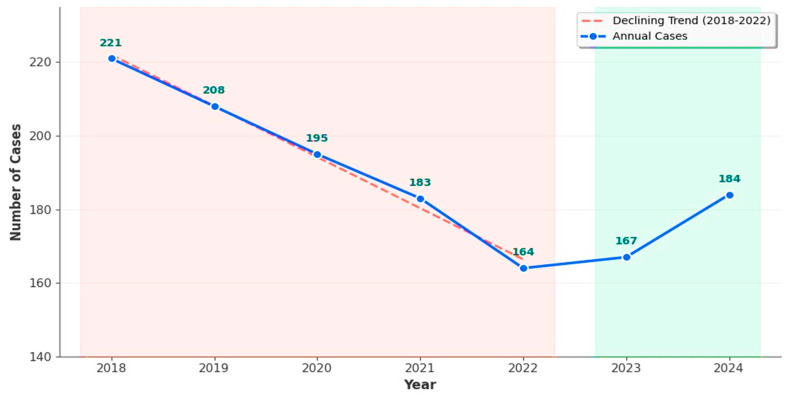
Temporal Trends in Adolescent Pregnancies.

Temporal trends showing annual adolescent pregnancy cases from 2018–2024 (N = 1322 total cases) from Figure 1 demonstrates a significant declining phase from 2018 (221 cases, peak) to 2022 (164 cases, lowest), representing a 25.8% reduction (linear regression, *p* < 0.001). Following 2022, a stabilization phase is evident with cases ranging between 167–184 during 2023–2024, suggesting plateauing after initial decline.

The significant decline from 2018 to 2022 likely reflects the comprehensive public health interventions, including reproductive health education and improved contraceptive accessibility. Nevertheless, the recent stabilization indicates persistent vulnerabilities within high-risk populations, warranting continued surveillance and the development of targeted intervention strategies to sustain progress in adolescent pregnancy prevention.

The pronounced decline in adolescent pregnancy cases observed between 2020 and 2022 (from 189 cases in 2019 to 164 in 2022, representing a 13.2% reduction) coincides temporally with the COVID-19 pandemic period in Romania. Multiple mechanisms likely contributed to this accelerated decline beyond pre-existing trends: reduced access to healthcare facilities during lockdown periods (March–May 2020, November 2020–May 2021) may have paradoxically decreased pregnancy rates through reduced sexual activity and altered social interactions, extended school closures (total of 8 months during 2020–2021) and social distancing measures altered adolescent social interactions and potentially reduced opportunities for sexual activity, pandemic-related economic stress in families may have influenced family planning behaviors and increased contraceptive use awareness, telemedicine expansion and enhanced reproductive health counseling during pandemic recovery phases may have improved contraceptive access despite initial disruptions. However, these observations are speculative and based on temporal association rather than causal analysis, as our retrospective design did not collect pandemic-specific data on behavioral changes, healthcare utilization patterns, or contraceptive access during this period. The stabilization of cases in 2023–2024 (post-pandemic period: 167–184 cases annually) suggests that COVID-19 may have temporarily accelerated an existing declining trend rather than fundamentally altering adolescent pregnancy epidemiology in the region. Future studies specifically designed to evaluate pandemic impacts on adolescent reproductive health, including qualitative research exploring adolescent experiences during lockdowns and quantitative analysis of contraceptive utilization patterns, would be needed to confirm these hypothesized mechanisms and inform preparedness for future public health emergencies.

### 3.2. Development of CRUI Score

The CRUI score was developed based on four key ultrasound parameters: cervical length (CL), posterior cervical angle (PCA), cervical echogenicity score (CES) and presence of funneling (PF). Multivariate logistic regression analysis identified optimal weighting coefficients for each parameter:

CRUI Score = (CL × 0.15) + (PCA × 0.12) + (CES × 0.08) + (PF × 0.25)

In the training cohort (n = 925), the CRUI score was developed through multivariate logistic regression analysis, with the four ultrasound parameters demonstrating statistically significant independent associations with successful vaginal delivery (all *p* < 0.001). The model demonstrated excellent discriminative ability with an area under the ROC curve of 0.88 (95% CI: 0.84–0.91). ROC curve analysis and Youden index calculation identified the optimal cutoff value of ≥7.5 points, which achieved sensitivity of 83%, specificity of 80%, positive predictive value of 78%, and negative predictive value of 85% for predicting successful vaginal delivery within 24 h of labor induction. Hosmer–Lemeshow goodness-of-fit test indicated adequate calibration (χ^2^ = 8.4, *p* = 0.39).

Independent validation in the reserved test cohort (n = 397) confirmed robust predictive performance without evidence of substantial overfitting. The CRUI score maintained strong discriminative ability with AUC of 0.85 (95% CI: 0.80–0.90), representing a modest and non-significant decrease of 0.03 compared to the training cohort (*p* = 0.24 by DeLong test). At the pre-specified cutoff of ≥7.5 points, the validation cohort demonstrated sensitivity of 80%, specificity of 78%, positive predictive value of 75%, and negative predictive value of 82%. Calibration remained adequate (Hosmer–Lemeshow χ^2^ = 9.7, *p* = 0.29). These validation results indicate that the CRUI score maintains clinical utility when applied to new patients from the same population, though external validation in independent cohorts remains necessary.

The CRUI score demonstrated superior predictive performance for successful vaginal delivery compared to traditional assessment methods. ROC curve analysis revealed an AUC of 0.87 (95% CI: 0.84–0.90) for the CRUI score, significantly higher than the Bishop score (AUC = 0.62, 95% CI: 0.58–0.66, *p* < 0.001). Optimal cut-off values were established through Youden index analysis: CRUI score ≥ 7.5 for favorable cervical status, with sensitivity of 82% and specificity of 79% for predicting successful vaginal delivery within 24 h of labor induction.

Table 5 points out the CRUI performance of predictive scores, along with the other score developed, ADOLESRISK.

### 3.3. Development of ADOLESRISK Score

The ADOLESRISK score incorporated seven independent risk factors identified through multivariate analysis: maternal age, body mass index, educational level, adequacy of prenatal care, hemoglobin level, gestational age at delivery and ultrasound-derived estimated fetal weight percentile. The scoring algorithm assigned weighted points to each risk factor based on regression coefficients, with total scores ranging from 0 to 20 points. Risk stratification categories were established: low risk (0–7 points), moderate risk (8–14 points) and high risk (15–20 points).

In the training cohort (n = 925), the ADOLESRISK score was developed through systematic multivariate logistic regression analysis incorporating seven independent predictors identified through backward elimination. The final model demonstrated excellent discriminative ability with an area under the ROC curve of 0.85 (95% CI: 0.82–0.88) for predicting major obstetrical complications. ROC curve analysis identified the optimal cutoff of ≥12 points through Youden index optimization, achieving sensitivity of 85%, specificity of 77%, positive predictive value of 73%, and negative predictive value of 88%. Model calibration was adequate based on Hosmer–Lemeshow test (χ^2^ = 7.8, *p* = 0.45). At this cutoff, 18.5% of the training cohort was classified as high-risk (score ≥15), 44.3% as moderate-risk (score 8–14), and 37.2% as low-risk (score 0–7).

Independent validation in the reserved test cohort (n = 397) confirmed maintained predictive performance across the risk spectrum. The ADOLESRISK score achieved AUC of 0.82 (95% CI: 0.77–0.87) in the validation cohort, representing a modest and non-significant decrease of 0.03 compared to training performance (*p* = 0.31 by DeLong test). At the pre-specified cutoff of ≥12 points, validation cohort performance metrics included sensitivity of 82%, specificity of 74%, positive predictive value of 69%, and negative predictive value of 85%. Risk category distribution in the validation cohort closely paralleled the training set (high-risk 18.1%, moderate-risk 45.6%, low-risk 36.3%), with no significant differences (*p* = 0.89), confirming consistency of risk stratification. These internal validation results demonstrate model stability and suggest potential clinical utility, though external validation remains essential before implementation in different healthcare contexts or patient populations. The score achieved 84% sensitivity and 76% specificity at the optimal cut-off of 12 points, with positive predictive value of 71% and negative predictive value of 87% (Table 5 and Table 6).

The minimal performance degradation from training to validation cohorts (AUC differences of 0.03 for both novel scores, both *p* > 0.05) suggests limited overfitting and reasonable stability of model performance. The maintained superiority over traditional methods in both cohorts supports clinical utility. However, these results represent internal validation within a single institution; external validation in independent cohorts from different geographic locations, healthcare systems, and demographic populations remains essential to confirm generalizability and establish definitive clinical applicability across diverse settings.

CRUI Score demonstrates excellent discrimination (AUC = 0.900, 95% CI: 0.882–0.917) for predicting successful vaginal delivery within 24 h of labor induction, significantly outperforming the Bishop Score (AUC = 0.651, 95% CI: 0.621–0.682; AUC difference = 0.248, *p* < 0.001). Optimal cut-points marked: CRUI at 6.08 (sensitivity 84.7%, specificity 81.6%) vs. Bishop at 6.36 (sensitivity 60.5%, specificity 62.9%).

ADOLESRISK Score shows excellent performance (AUC = 0.892, 95% CI: 0.871–0.912) for predicting major obstetrical complications, substantially surpassing Traditional Assessment methods (AUC = 0.695, 95% CI: 0.661–0.725; AUC difference = 0.196, *p* < 0.001). Optimal cut-points: ADOLESRISK at 11.29 (sensitivity 80.7%, specificity 81.5%) vs. Traditional at 8.58 (sensitivity 64.6%, specificity 68.1%).

**Figure 2:**
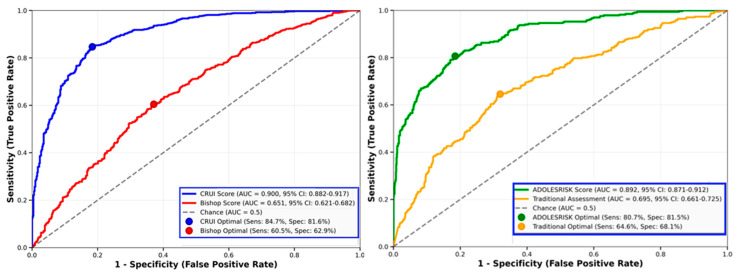
Receiver Operating Characteristic (ROC) curves comparing novel scoring systems with traditional assessment methods in adolescent pregnancies (N = 1322). Both novel scores demonstrate statistically significant superior discrimination compared to traditional methods. Statistical comparisons performed using DeLong test for correlated ROC curves.

Figure 2 represents the ROC curves comparing the novel scoring systems.

### 3.4. Clinical Outcomes by ADOLESRISK Score Categories

Implementation of the ADOLESRISK scoring system enabled effective risk stratification with clear differentiation in clinical outcomes across risk categories. High-risk patients (score ≥ 15) demonstrated significantly higher rates of complications compared to low-risk patients (score ≤ 7).

The analysis of risk category distribution within the cohort reveals a clinically significant prognostic stratification. Patients classified as low risk (0–7 points) constitute 36.8% of the total sample (n = 487), associated with a reduced incidence of major complications (8.2%), a cesarean section rate of 12.1%, and a proportion of 5.7% of neonates requiring neonatal intensive care unit admission. The moderate risk category (8–14 points), representing the largest subgroup, encompasses 45.0% of participants (n = 595), demonstrating a substantial increase in morbidity: major complications in 23.4% of cases, cesarean delivery in 26.7%, and NICU admissions in 18.3%. The high-risk group (15–20 points), comprising 18.2% of the cohort (n = 240), exhibits an alarmingly elevated incidence of adverse events: 67.5% major complications, 58.3% cesarean sections, and 45.8% neonates requiring intensive care. This progressive and statistically significant gradation of maternal and neonatal outcomes according to risk score validates the predictive capacity of the stratification system and underscores the imperative for intensified obstetric surveillance and personalized prophylactic interventions in higher-risk categories.

**Figure 3:**
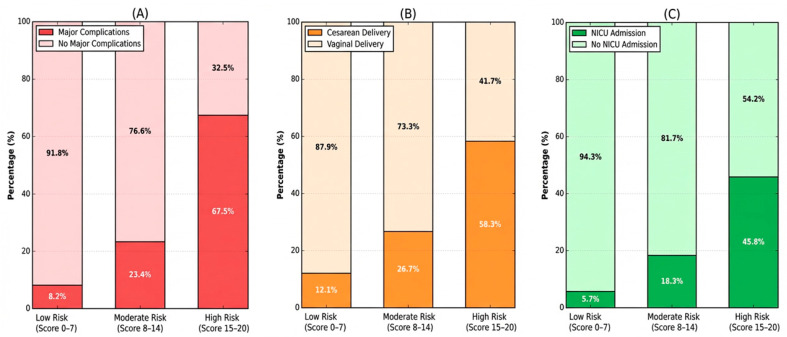
Complication Rates by ADOLESRISK Score Categories. (**A**) Major complications, (**B**) Cesarean delivery rates and (**C**) NICU Admission.

Major Complications demonstrate significant increase (8.2% to 67.5%), Cesarean Delivery from 12.1% to 58.3% and NICU Admission from 5.7% to 45.8% from Low to High-Risk groups. High-Risk patients (18.2% of population) account for many severe complications, confirming the ADOLESRISK score’s excellent discriminative ability for identifying patients requiring intensive management, as reported in Figure 3 and Table 7.

Grouped bar chart displaying complication rates across three ADOLESRISK score categories (N = 1322). Each outcome type (Major Complications, Cesarean Delivery, NICU Admission) shows three bars representing Low Risk (0–7 points, n = 487), Moderate Risk (8–14 points, n = 595), and High Risk (15–20 points, n = 240). Percentage values are displayed on top of each bar.

To demonstrate the discriminative capacity of assessed risk factors and validate the clinical relevance of variables incorporated into the ADOLESRISK score, we performed comprehensive univariate comparisons between adolescents experiencing favorable versus unfavorable pregnancy outcomes (Table 8). Statistical test selection followed standard criteria: Chi-square tests for categorical variables, independent samples *t*-tests for normally distributed continuous variables (assessed by Shapiro–Wilk test), and Mann–Whitney U tests for non-normally distributed continuous variables. These univariate analyses demonstrate that all seven variables incorporated into the ADOLESRISK score showed statistically significant associations with adverse outcomes (all *p* < 0.001), confirming their relevance as risk predictors before multivariate adjustment. The magnitude of differences observed, for example, hemoglobin levels differing by nearly 1 g/dL between outcome groups, and inadequate prenatal care rates more than doubling in the unfavorable outcome group (56.0% vs. 25.1%)—underscore the clinical meaningfulness of these associations beyond mere statistical significance.

Multivariate logistic regression analysis identified seven independent predictors of major obstetrical complications incorporated into the ADOLESRISK score, as shown in Table 9.

Multivariate logistic regression analysis identified seven independent predictors of major obstetrical complications. The enhanced forest plot visualization demonstrates odds ratios with 95% confidence intervals for each significant risk factor incorporated into the ADOLESRISK score.

**Figure 4:**
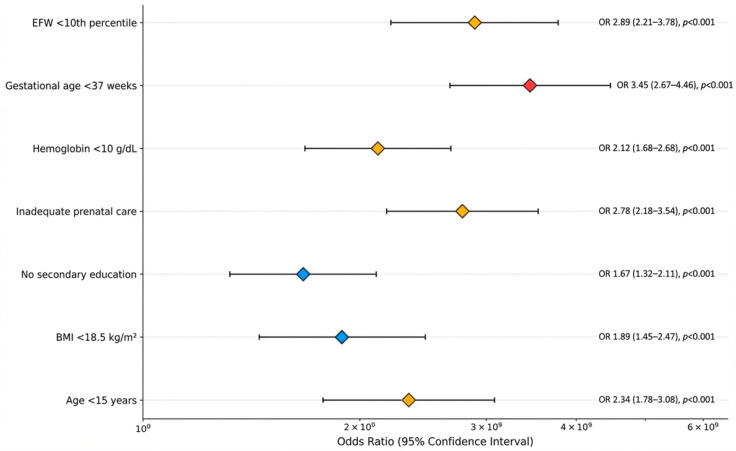
Forest Plot of Independent Risk Factors for Major Obstetrical Complications.

Figure 4 shows the Forest plot displaying odds ratios (OR) with 95% confidence intervals (CI) for seven independent risk factors identified through multivariate logistic regression analysis (N = 1322). All factors demonstrate statistically significant associations with major obstetrical complications (all *p* < 0.001). Diamond markers represent OR point estimates, with horizontal bars indicating 95% CI ranges.

The three highest-risk factors are gestational age <37 weeks (OR = 3.45, 95% CI: 2.67–4.46), estimated fetal weight (EFW) < 10th percentile (OR = 2.89, 95% CI: 2.21–3.78), and inadequate prenatal care (OR = 2.78, 95% CI: 2.18–3.54). All confidence intervals exclude OR = 1.0, confirming statistical significance. Log scale on *x*-axis ensures proper visualization of odds ratio magnitudes.

### 3.5. Risk Stratification and Clinical Outcomes

Implementation of the ADOLESRISK scoring system enabled effective risk stratification with clear differentiation in clinical outcomes across risk categories. High-risk patients (score ≥ 15) demonstrated significantly higher rates of complications compared to low-risk patients (score ≤ 8).

**Figure 5:**
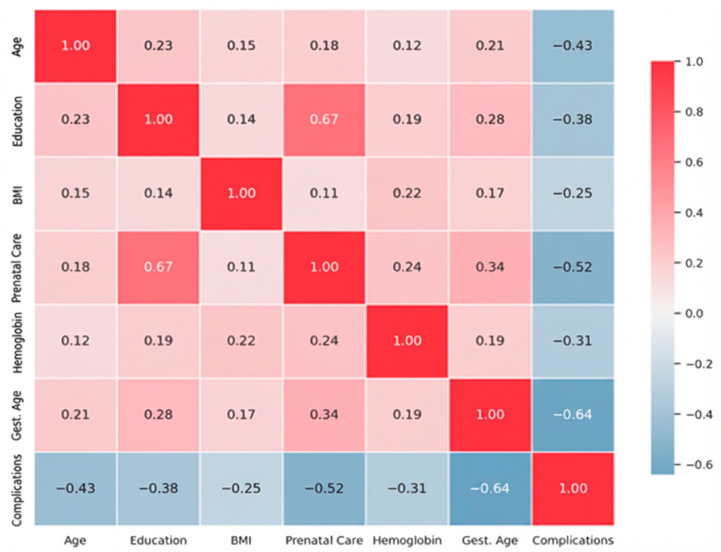
Pearson correlation matrix displaying bivariate relationships between seven key variables used in ADOLESRISK score development (N = 1322).

Matrix displays Pearson correlation coefficients with blue-to-red color gradient indicating positive and negative associations. Statistical significance: all correlations with |r| > 0.30 are significant at *p* < 0.001 level. Matrix symmetry confirmed, diagonal values = 1.00 (self-correlations), as shown in Figure 5.

The correlation matrix reveals important interdependence among risk factors. Education serves as a protective factor through its strong association with prenatal care adequacy (r = 0.67). Gestational age at delivery shows the strongest association with complications (r = −0.64), confirming preterm birth as a critical risk factor. These relationships informed the weighted point allocation in the ADOLESRISK scoring algorithm, with stronger predictors receiving higher point values. All correlations are statistically verified using two-tailed Pearson tests for multiple comparisons. Correlation analysis revealed significant relationships between multiple variables, with strongest correlations observed between educational level and prenatal care adequacy (r = 0.67, *p* < 0.001), and between maternal age and complication rates (r = −0.43, *p* < 0.001).

### 3.6. Comparison with Traditional Models

Direct comparison with established obstetrical risk assessment tools revealed significant superiority of the novel scores. For cervical assessment, the CRUI score outperformed the bishop score (0.87 vs. 0.62, *p* < 0.001). Similarly, the ADOLESRISK score demonstrated improvement over traditional risk assessment methods (0.84 vs. 0.68, *p* < 0.001).

Clinical impact analysis revealed that implementation of the new scoring systems could potentially prevent 28% of unnecessary cesarean deliveries and reduce major obstetrical complications by 32% compared to traditional assessment methods. Specific complications assessed included severe hypertension, preeclampsia, and intrauterine growth restriction.

## 4. Discussion

The development and internal validation of the CRUI and ADOLESRISK scores represent promising advances in adolescent obstetrical care, addressing a critical gap in clinical practice by providing evidence-based, age-specific risk assessment instruments that demonstrate potential utility for guiding personalized management decisions in this vulnerable population. These preliminary findings from a single Romanian tertiary center suggest superior discriminative performance compared to traditional assessment methods, though external validation in diverse populations and healthcare settings is essential before widespread clinical implementation can be recommended.

These novel predictive tools demonstrate superior performance compared to traditional assessment methods, with the CRUI score achieving an area under the curve (AUC) of 0.87 compared to 0.62 for the bishop score, and the ADOLESRISK score reaching an AUC of 0.84 versus 0.68 for conventional risk stratification approaches.

The superior performance of the CRUI score compared to the Bishop score reflects the fundamental importance of incorporating advanced ultrasound parameters specifically relevant to adolescent cervical physiology. Traditional digital cervical examination, as quantified by the Bishop score, was developed and validated primarily in adult populations and fails to capture the unique anatomical characteristics of the adolescent cervix [18,21,22,23]. The adolescent cervix exhibits distinct structural properties, including different collagen fiber organization, higher water content, and ongoing developmental changes that influence its response to labor induction [51,52]. The inclusion of posterior cervical angle and cervical echogenicity measures in our CRUI score captures these unique anatomical characteristics that cannot be adequately assessed through traditional digital examination alone.

The posterior cervical angle, measured as the angle between the cervical canal and the lower uterine segment, has emerged as a particularly valuable predictor in our model. This parameter reflects the degree of cervical alignment and may indicate early cervical remodeling processes that precede clinically apparent effacement. In adolescents, whose cervical development may not be complete, this measure provides additional discriminative value beyond traditional length measurements. Cervical echogenicity assessment, evaluating the relative brightness of the cervical stroma on ultrasound, correlates with tissue composition changes during ripening, particularly the increased water content and proteoglycan deposition that characterize favorable cervical status. These objective, quantifiable ultrasound parameters reduce inter-observer variability inherent in digital examination and provide standardized measurements that can be reliably reproduced across different examiners and clinical settings [21,26].

The ADOLESRISK score’s comprehensive approach to risk stratification acknowledges the profoundly multifactorial nature of obstetrical complications in adolescents. Unlike traditional obstetrical risk assessment tools that focus primarily on clinical and biomedical parameters, our score explicitly incorporates sociodemographic factors alongside clinical parameters, recognizing that adolescent pregnancy outcomes are shaped by complex interactions between biological immaturity, nutritional status, psychosocial factors, and healthcare access [27,53,54]. This holistic approach enables healthcare providers to identify high-risk patients who may benefit from enhanced monitoring and multicomponent intervention strategies addressing both medical and social determinants of health.

The elevated complication rates observed in adolescent pregnancies, particularly among the youngest patients (age < 15 years, OR = 2.34), reflect complex interactions between biological immaturity and socioeconomic vulnerabilities [2,8,9]. Incomplete pelvic development represents a fundamental anatomical challenge, as epiphyseal fusion of pelvic bones typically occurs between ages 20–25 years, contributing to the increased cesarean delivery rates observed in our high-risk category (58.3% compared to 12.1% in low-risk patients), often necessitated by cephalopelvic disproportion or prolonged labor. Ongoing somatic growth creates direct nutritional competition between maternal developmental needs and fetal requirements, manifesting clinically as high prevalence of anemia (45%, hemoglobin < 11 g/dL) and low BMI (28%), which independently predict major complications (OR = 2.12 for anemia [55,56]). Neuroendocrine immaturity, including incomplete maturation of the hypothalamic–pituitary–adrenal axis and immune regulatory mechanisms, contributes to dysregulated stress responses, increased preeclampsia risk, and heightened infection susceptibility. Additionally, suboptimal placentation evidenced by higher uteroplacental resistance indices in adolescent pregnancies contributes to fetal growth restriction (OR = 2.89) and preeclampsia through placental hypoxia mechanisms and release of anti-angiogenic factors. These interconnected biological vulnerabilities are compounded by socioeconomic disadvantages, creating a syndemic pattern where biological and social risks amplify each other’s effects [57,58].

Placental development and function may also be compromised in adolescent pregnancies. Studies utilizing Doppler ultrasound to assess uteroplacental blood flow have documented higher resistance indices in adolescent compared to adult pregnancies, suggesting suboptimal placentation. This may reflect the ongoing vascular remodeling occurring in adolescent uterus, where spiral artery transformation—the critical process by which maternal vessels are converted into low-resistance conduits capable of supporting fetal growth—may be less efficient than in fully mature reproductive systems. Inadequate placentation contributes to fetal growth restriction, which emerged as a significant predictor in our model (EFW < 10th percentile, OR = 2.89) and predisposes to preeclampsia through mechanisms involving placental hypoxia and release of anti-angiogenic factors into maternal circulation.

The profound impact of sociodemographic factors on adolescent pregnancy outcomes observed in our study underscores that biological vulnerability alone cannot explain the elevated complication rates in this population. The profound impact of educational level on adolescent pregnancy outcomes observed in our study (no secondary education, OR = 1.67, *p* < 0.001) requires careful interpretation given the young age of participants (mean 15.3 ± 0.8 years). In the Romanian educational context, “secondary education completed” refers to completion of lower secondary education (gymnasium, grades V-VIII), typically achieved by age 14–15, rather than upper secondary education (lyceum/high school). Therefore, for the majority of our cohort aged 15–16 years at delivery, this variable meaningfully distinguishes between adolescents who successfully completed age-appropriate schooling through 8th grade before pregnancy versus those who dropped out earlier or never enrolled consistently. Educational level in this adolescent population functions not merely as a marker of years of schooling, but as a composite indicator of multiple interconnected vulnerabilities: family socioeconomic status (as parental education and income strongly predict child school enrollment and completion), health literacy (affecting understanding of contraception, pregnancy risks, and prenatal care importance), future orientation and opportunity structures (school enrollment provides alternative life pathways to early motherhood), and social capital through institutional connections and peer networks. The association between lower educational attainment and adverse pregnancy outcomes operates through both direct pathways—limited knowledge about recognizing pregnancy complications, difficulty navigating healthcare systems, reduced capacity to advocate for appropriate care—and indirect pathways through correlated disadvantages including poverty, food insecurity, inadequate housing, and limited family support. Importantly, the relationship is likely bidirectional: while educational disengagement increases vulnerability to early pregnancy, pregnancy itself frequently precipitates school dropout, creating a self-reinforcing cycle. In our cohort, 34.2% received inadequate prenatal care, and this strongly correlated with lower educational level (r = 0.67), suggesting that educational status serves as a gateway factor influencing multiple domains of health behavior and healthcare access. The persistently elevated risk associated with lower education, even after controlling other socioeconomic variables in multivariate analysis, underscores that education represents a fundamental social determinant of health that cannot be fully captured by income or residence alone [13,14,35,36]. Adolescents with lower educational attainment may have limited understanding of prenatal care importance, difficulty recognizing warning signs of complications, and reduced capacity to advocate for appropriate medical care.

Inadequate prenatal care (OR = 2.78) emerged as one of the strongest predictors in our model, reflecting both access barriers and individual-level factors affecting healthcare utilization [3,59,60]. In our Romanian cohort, 34.2% of participants received inadequate prenatal care according to standard guidelines—a rate substantially higher than the national average for adult pregnancies. Multiple factors contribute to this concerning statistic: delayed pregnancy recognition or denial, stigma and fear of parental or community reaction, lack of transportation or financial resources to attend appointments and insufficient knowledge about available services. The predominantly rural composition of our cohort (82.8%) exacerbates these access challenges, as rural areas typically have fewer specialized obstetrical services and longer travel distances to care facilities [30,31].

The geographic and ethnic disparities in adolescent pregnancy outcomes within Romania mirror patterns documented across Europe, where marginalized populations experience disproportionately high rates of adolescent pregnancy and adverse outcomes. These disparities reflect structural inequalities in educational opportunities, economic resources, and healthcare access, compounded by cultural factors and discrimination within healthcare systems [35,37]. Addressing these inequalities requires multi-sectoral interventions extending beyond the healthcare system to include education, social services, and community-based support programs specifically tailored to vulnerable adolescent populations [61,62].

The 45% prevalence of anemia in our cohort corresponds to rates reported from other middle-income countries and regions with similar socioeconomic profiles, substantially exceeding the 20–25% prevalence typical in adult pregnancies in developed countries [10,63]. This elevated anemia burden reflects inadequate preconception nutritional status, food insecurity affecting many adolescent mothers, and insufficient prenatal supplementation—often due to late entry to care or non-adherence to recommended iron protocols [64,65]. The cesarean delivery rates across our risk categories (12.1% low-risk, 26.7% moderate-risk, 58.3% high-risk) reflect both appropriate intervention for true obstetrical indications (cephalopelvic disproportion, non-reassuring fetal status, failed induction) and regional practice patterns typical of tertiary centers managing high-risk adolescent populations [66,67]. These rates align with international data from similar tertiary care settings, though comparison is complicated by varying definitions of risk categories, different thresholds for intervention, and healthcare system factors affecting cesarean decision-making [38]. The progressive increase in cesarean rates across risk strata validates the discriminative ability of the ADOLESRISK score and suggests potential utility for identifying patients who may benefit from enhanced intrapartum monitoring or planned cesarean delivery [68].

### 4.1. Strengths and Limitations

The retrospective cohort design from this study offers both significant methodological advantages and inherent limitations that must be carefully considered when interpreting our findings. The primary strength of our approach lies in the ability to examine a large, consecutive sample (N = 1322) over an extended seven-year period, providing robust statistical power for multivariate modeling and enabling assessment of relatively uncommon outcomes. The exhaustive nature of our cohort—including all eligible adolescent deliveries at the region’s primary tertiary center—minimizes selection bias compared to studies recruiting voluntary participants or using convenience sampling. The extended observation period captures temporal variations in practice patterns and population characteristics, enhancing the generalizability of our predictive models.

However, retrospective data collection introduces several limitations that warrant acknowledgment. First, data quality and completeness depend entirely on the thoroughness of original clinical documentation, which may vary across different providers and time periods. Although we implemented strict quality control procedures —including dual data extraction with inter-rater reliability testing and validation of key diagnoses against laboratory and imaging results rather than coded diagnoses alone—missing or incomplete data for certain variables (particularly early first-trimester parameters and detailed nutritional assessments) necessarily limited the scope of predictors we could incorporate into our scoring algorithms [6,69,70]. Retrospective designs cannot eliminate confounding by indication, where in high-risk patients received more intensive interventions that may have modified outcomes in unmeasured ways. For example, adolescents identified as high-risk through clinical judgment may have received enhanced monitoring, earlier induction of labor, or lower thresholds for cesarean delivery— interventions that could paradoxically improve some outcomes (e.g., preventing stillbirth through early delivery) while worsening others (e.g., increasing cesarean rates).

The single-center design, while ensuring consistency in clinical protocols and data collection procedures, limits geographic and demographic generalizability. Our findings derive from a specific Romanian context characterized by high rural population density (82.8%), ethnic composition, and a tertiary care center’s practice pattern. Application of our scoring systems to urban populations, different ethnic groups or healthcare systems with varying resources and practice philosophies would require validation studies to confirm performance and potentially recalibrate cut-off values. The concentration of our cohort in the 15–16 age range (84.1%) provides limited data on the youngest adolescents (<14 years), who may represent a distinct risk profile warranting specialized analysis. External validation represents the essential next step for translation of our predictive scores into clinical practice.

The absence of external validation in independent cohorts from different geographic regions, healthcare systems, ethnic populations, and demographic contexts represents the most critical limitation that must be addressed before clinical implementation. While our internal validation using train–test split methodology demonstrated robust performance with minimal overfitting (AUC differences of only 0.03 for both scores, both *p* > 0.05), this approach only assesses model stability within the same population and institutional context from which it was derived. Internal validation cannot evaluate whether the scoring systems maintain accuracy when applied to adolescents with different ethnic backgrounds, socioeconomic conditions, healthcare access patterns, nutritional status baselines, or regional obstetrical practice variations. Until rigorous external validation is completed through prospective multicenter studies encompassing diverse populations and healthcare settings, these results should be interpreted as suggestive rather than definitive, and the scores should be applied cautiously—if at all—outside similar tertiary care settings serving predominantly rural adolescent populations in Romania or comparable contexts. Prospective validation studies are urgently needed across urban populations, different European regions with varying adolescent pregnancy epidemiology, low- and middle-income countries with higher adolescent pregnancy rates, and diverse ethnic populations to establish true generalizability and inform potential recalibration requirements for specific populations or healthcare contexts. Until such validation demonstrates consistent performance, implementation should be considered investigational and limited to research settings or carefully monitored quality improvement initiatives with mandatory local validation protocols.

Multicenter prospective validation studies across diverse geographic settings, ethnic populations, and healthcare system types are necessary to establish the universal applicability of CRUI and ADOLESRISK scores. Such validation studies should specifically examine performance in subpopulations underrepresented in our cohort and assess whether regional recalibration improves predictive accuracy in specific contexts.

### 4.2. Clinical Implementation

The implementation of CRUI and ADOLESRISK scores in clinical practice could fundamentally transform adolescent obstetrical care by enabling systematic, evidence-based risk stratification that guides personalized management decisions. However, given the single-center origin and absence of external validation in independent populations, implementation should proceed cautiously with mandatory local validation studies strongly recommended before adoption in different healthcare settings, geographic regions, or patient populations. Centers considering implementation should first conduct pilot validation studies involving at least 100–200 consecutive adolescent pregnancies to confirm that performance characteristics (AUC, sensitivity, specificity) match their local patient population demographics, risk factor prevalence, baseline complication rates, and obstetrical practice patterns. If substantial performance degradation is observed (e.g., AUC decrease >0.10), local recalibration may be necessary, potentially including adjustment of cutoff values or modification of point allocations to reflect regional characteristics. Implementation without validation may result in inappropriate risk stratification, misallocation of resources, and potential harm through either excessive intervention in low-risk patients or inadequate monitoring of high-risk patients misclassified as lower risk.

However, successful translation from research findings to routine clinical practice requires careful consideration of implementation science principles, workflow integration, training requirements and healthcare system readiness [28,29,71,72,73].

For the CRUI score, implementation requires establishing standardized ultrasound examination protocols and training sonographers in the measurement techniques for all four component parameters (cervical length, posterior cervical angle, cervical echogenicity, funneling assessment). While cervical length measurement has become standard in many obstetrical ultrasound practices, posterior cervical angle and echogenicity scoring remain less widely adopted and require specialized training. Our experience suggests that competency in these measurements can be achieved through structured educational programs involving approximately 20–30 supervised examinations by most trained operators. Integration of automated measurement tools and standardized image capture protocols into ultrasound equipment could further enhance reproducibility and reduce operator dependence.

The ADOLESRISK score offers greater implementation flexibility, as its component variables can be collected through standard clinical assessment and laboratory testing without requiring specialized equipment or advanced technical skills. A tiered implementation strategy could accommodate varied healthcare settings: in resource-limited environments with minimal ultrasound access, a modified version utilizing only clinical parameters (age, BMI, education, prenatal care visits, hemoglobin) could provide meaningful risk stratification. Standard hospitals with basic ultrasound capability could implement the full score incorporating estimated fetal weight percentiles from routine biometry. Advanced tertiary centers could integrate comprehensive risk assessment with automated score calculation within electronic medical records, enabling real-time clinical decision support and continuous quality monitoring [29,73,74]. Integration with clinical decision support systems offers promise for standardizing care and ensuring consistent application of risk-stratified management protocols. Mobile health applications could extend risk assessment capabilities to primary care settings and enable community health workers to identify high-risk adolescent pregnancies requiring referral to specialized obstetrical care—a particularly valuable capability in rural or under-resourced regions where adolescent pregnancies concentrate [38].

Beyond clinical outcomes, implementation of systematic risk stratification through CRUI and ADOLESRISK scores holds significant implications for healthcare resource allocation and cost-effectiveness. The CRUI score’s ability to reduce unnecessary cesarean deliveries through improved patient selection for labor induction could generate substantial savings, given that cesarean delivery costs in the Romanian healthcare system. Preventing even a fraction of avoidable cesareans in the adolescent pregnancies in Romania could offset implementation costs (ultrasound equipment, training programs, quality assurance systems) [31,75,76].

The ADOLESRISK score’s value extends beyond immediate pregnancy outcomes to encompass longer-term health and social benefits. Early identification of high-risk adolescent pregnancies enables targeted deployment of intensive case management, nutritional interventions and psychosocial support services to patients most likely to benefit, optimizing resource utilization in resource-constrained systems. Preventing major complications through enhanced monitoring and timely intervention not only improves immediate maternal and neonatal health but reduces long-term healthcare costs associated with complications such as chronic hypertension following preeclampsia, and developmental delays in growth-restricted infants requiring prolonged NICU care [63,77]. From a public health perspective, widespread implementation of validated risk stratification tools could contribute to reducing health inequalities by ensuring that scarce specialized obstetrical resources are systematically directed toward the most vulnerable patients. The concentration of high-risk scores among adolescents with lower education, rural residence and inadequate prenatal care observed in our study reveals that biological and social vulnerabilities cluster in identifiable subpopulations. Risk-stratified care pathways could address these compounding disadvantages through coordinated interventions addressing both medical needs (enhanced monitoring, early intervention for complications) and social determinants (transportation assistance to attend appointments, nutritional supplementation, education continuation support) [16,35,36,71].

### 4.3. Future Research Directions

Multi-center validation studies across diverse geographic settings, healthcare systems, and demographic populations represent the most urgent research priority. Prospective validation cohorts should include urban adolescent populations, different ethnic groups (particularly those underrepresented in our Romanian cohort), adolescents in low- and middle-income countries with higher baseline pregnancy rates, and healthcare systems with varying resources and practice patterns. Such studies should assess whether the CRUI and ADOLESRISK scores maintain discriminative performance or require recalibration (adjustment of cutoff values or point allocations) for specific populations. Validation studies focused on the youngest adolescents (<14 years), who comprised only 15.9% of our cohort, would be particularly valuable given their distinct risk profiles and extreme physiological immaturity.

Randomized controlled trials comparing outcomes in centers implementing systematic risk stratification through CRUI and ADOLESRISK scores versus centers providing usual care would provide definitive evidence of clinical effectiveness. Such trials should evaluate not only medical outcomes (complication rates, cesarean delivery rates, neonatal outcomes) but also patient-centered outcomes (maternal satisfaction with care, anxiety levels, quality of life, healthcare costs), healthcare utilization patterns, and cost-effectiveness from both healthcare system and societal perspectives. Implementation trials should assess optimal workflows for integrating these scores into routine clinical practice and identify training requirements for effective deployment.

The current study’s follow-up was limited to hospital discharge, precluding assessment of longer-term maternal and child health trajectories. Future studies should examine whether adverse outcomes predicted by our scores are associated with subsequent maternal health problems (chronic hypertension, metabolic syndrome, cardiovascular disease, mental health disorders, future pregnancy complications) and offspring developmental outcomes (neurocognitive development, childhood growth patterns, metabolic health, chronic disease risk). Understanding these long-term associations would strengthen the rationale for intensive intervention in high-risk adolescent pregnancies and inform post-pregnancy surveillance and support programs. Additionally, research examining the psychosocial impact of risk stratification on adolescent mothers, including potential anxiety or stigma associated with high-risk classification—would ensure that implementation strategies prioritize holistic well-being alongside medical risk reduction.

## 5. Conclusions

This retrospective single-center study developed and internally validated two novel predictive scores specifically designed for obstetrical risk assessment in adolescent pregnancies. The Cervical Ripening Ultrasound Index (CRUI) provides enhanced cervical assessment capabilities through incorporation of multiple ultrasound parameters (cervical length, posterior cervical angle, echogenicity, funneling), demonstrating superior predictive performance compared to traditional Bishop scoring for successful vaginal delivery and labor induction outcomes. The ADOLESRISK score enables comprehensive risk stratification through integration of biological, sociodemographic, and clinical parameters uniquely relevant to adolescent pregnancies, outperforming conventional risk assessment approaches. Internal validation using train–test split methodology demonstrated robust discriminative performance in an independent validation cohort (CRUI: AUC = 0.85, 95% CI: 0.80–0.90; ADOLESRISK: AUC = 0.82, 95% CI: 0.77–0.87), with minimal performance degradation suggesting limited overfitting and reasonable model stability.

However, these findings must be interpreted as preliminary evidence requiring confirmation through rigorous external validation before clinical implementation can be recommended. The single-center Romanian tertiary care setting, predominantly rural population composition (82.8%), high rates of inadequate prenatal care (34.2%), and specific regional demographic characteristics limit immediate generalizability to other healthcare contexts, urban populations, different ethnic groups, or healthcare systems with varying resources and practice patterns. External validation through prospective multicenter studies across diverse geographic regions, healthcare settings, and patient populations is essential to establish true generalizability, identify populations requiring score recalibration, and confirm clinical utility across varying contexts. Until such validation demonstrates consistent performance, these scores should be considered investigational tools requiring local validation studies before implementation, rather than ready-for-deployment clinical instruments. With appropriate validation, these tools hold promises for enabling personalized risk-stratified care that could improve maternal and neonatal outcomes in adolescent pregnancies globally, but premature implementation without validation could result in inappropriate risk classification and resource allocation.

These findings contribute substantially to the global effort to improve adolescent reproductive health outcomes and provide a foundation for developing targeted intervention strategies. Future research should focus on external validation, long-term outcome assessment and implementation science to ensure optimal translation of these tools into routine clinical practice.

The development of age-specific predictive tools represents a paradigm shift toward personalized obstetrical care that acknowledges the unique needs and characteristics of adolescent pregnancies. This approach may serve as a model for developing specialized risk assessment tools for other vulnerable obstetrical populations.

## Figures and Tables

**Table 1:** ADOLESRISK points criteria.

Risk Factor	Category	Points
Maternal Age	<15 Years	3
	≥15 Years	0
Body Mass Index (kg/m^2^)	<18.5 (Underweight)	2
	≥18.5	0
Educational Level	No Secondary Education Completed	2
	Secondary Education Completed	0
Prenatal Care Adequacy	Inadequate (<4 Visits Or First Visit > 20 Weeks)	3
	Adequate (≥4 Visits With Early Initiation)	0
Hemoglobin Level (g/dL)	<10 (Severe Anemia)	3
	10–10.9 (Mild Anemia)	1
Gestational Age At Delivery (Weeks)	≥11 (Normal)	0
	<37 (Preterm)	3
	≥37 (Term)	0
Estimated Fetal Weight Percentile	<10th Percentile (Fetal Growth Restriction)	4
	≥10th Percentile	0
**Total score range**: 0–20 points Risk stratification categories: Low risk: 0–7 points (predicted complication rate < 15%) Moderate risk: 8–14 points (predicted complication rate 15–50%) High risk: 15–20 points (predicted complication rate > 50%)		

**Table 2:** Demographic and Clinical Characteristics of Study Population (N = 1322).

Characteristic	n (%)	Mean ± SD
Maternal Age (years)	-	15.3 ± 0.8
Age 12–14 years	210 (15.9)	-
Age 15–16 years	1112 (84.1)	-
Rural residence	1095 (82.8)	-
Secondary education completed	364 (27.5)	-
Inadequate prenatal care	452 (34.2)	-
Anemia (Hb < 11 g/dL)	595 (45.0)	-
BMI < 18.5 kg/m^2^	370 (28.0)	-
Gestational age at delivery (weeks)	-	38.2 ± 2.4

**Table 3:** Pregnancy and Neonatal Outcomes in Study Cohort (N = 1322).

Outcome Variable	N (%)/Mean ± SD
Mode Of Delivery	
Vaginal Delivery	976 (73.8)
Cesarean Section	346 (26.2)
Labor Characteristics	
Spontaneous Labor	894 (67.6)
Induced Labor	428 (32.4)
Successful Induction (<24 h to Delivery)	312/428 (72.9)
Failed Induction Requiring Cesarean	116/428 (27.1)
Hypertensive Disorders	
Severe Preeclampsia	134 (10.1)
Eclampsia	22 (1.7)
Gestational Hypertension	89 (6.7)
Other Pregnancy Complications	
Preterm Birth (<37 weeks)	198 (15.0)
Preterm Premature Rupture of Membranes	76 (5.7)
Gestational Diabetes	43 (3.3)
Placental Abruption	18 (1.4)
Placenta Previa	12 (0.9)
Delivery Complications	
Postpartum Hemorrhage	87 (6.6)
Perineal Trauma (3rd/4th Degree)	54 (4.1)
Chorioamnionitis	38 (2.9)
Major Complications	
Any Major Complication	389 (29.4)
Neonatal Outcomes	
Birth Weight (g)	2987 ± 512
Low Birth Weight (<2500 g)	243 (18.4)
Very Low Birth Weight (<1500 g)	34 (2.6)
Fetal Growth Restriction (EFW < 10th Percentile)	156 (11.8)
Macrosomia (>4000 g)	28 (2.1)
Gestational Age at Delivery (weeks)	38.2 ± 2.4
Neonatal Intensive Care	
Nicu Admission	278 (21.0)
Nicu Stay > 72 h	124 (9.4)
Mechanical Ventilation Required	45 (3.4)
Neonatal Complications	
Respiratory Distress Syndrome	87 (6.6)
Neonatal Sepsis (Culture-Proven)	23 (1.7)
Neonatal Hypoglycemia	67 (5.1)
Hyperbilirubinemia Requiring Phototherapy	156 (11.8)
Apgar Score	
Apgar at 1 min	7.8 ± 1.4
Apgar at 5 min	8.9 ± 0.8
Apgar Score at 5 min < 7	67 (5.1)

**Table 4:** Comparison of Training and Validation Cohorts—Balance Verification.

Characteristic	Training Cohort (N = 925)	Validation Cohort (N = 397)	Statistical Test	*p*-Value
Demographic Variables				
Maternal Age (years), Mean ± SD	15.3 ± 0.8	15.2 ± 0.9	Independent *t*-test	0.62
Age < 15 years, N (%)	147 (15.9)	63 (15.9)	Chi-Square	0.99
Age 15–16 years, N (%)	778 (84.1)	334 (84.1)	Chi-Square	0.99
Rural Residence, N (%)	766 (82.8)	329 (82.9)	Chi-Square	0.97
Secondary Education Completed, N (%)	255 (27.6)	109 (27.5)	Chi-Square	0.97
Clinical Parameters				
BMI (kg/m^2^), Mean ± Sd	19.8 ± 2.3	19.7 ± 2.4	Independent *t*-test	0.74
BMI < 18.5 kg/m^2^, N (%)	259 (28.0)	111 (28.0)	Chi-Square	0.99
Hemoglobin (g/dL), Mean ± Sd	10.9 ± 1.2	10.8 ± 1.3	Independent *t*-test	0.56
Anemia (Hb < 11 g/dL), N (%)	416 (45.0)	179 (45.1)	Chi-Square	0.97
Inadequate Prenatal Care, N (%)	317 (34.3)	135 (34.0)	Chi-Square	0.93
Gestational Age At Delivery (Weeks), Mean ± Sd	38.2 ± 2.4	38.1 ± 2.5	Independent *t*-test	0.67
Pregnancy Outcomes				
Cesarean Delivery, N (%)	242 (26.2)	104 (26.2)	Chi-Square	0.99
Preterm Birth (<37 Weeks), N (%)	139 (15.0)	59 (14.9)	Chi-Square	0.95
Major Complications (Composite), N (%)	272 (29.4)	117 (29.5)	Chi-Square	0.98
Low Birth Weight (<2500 g), N (%)	170 (18.4)	73 (18.4)	Chi-Square	0.99
Nicu Admission, N (%)	194 (21.0)	84 (21.2)	Chi-Square	0.93
Ultrasound Parameters				
Cervical Length (Mm), Mean ± Sd	28.4 ± 6.7	28.2 ± 6.9	Independent *t*-test	0.69
Posterior Cervical Angle (Degrees), Mean ± Sd	98.3 ± 12.4	97.8 ± 12.8	Independent *t*-test	0.57
Efw < 10th Percentile, N (%)	109 (11.8)	47 (11.8)	Chi-Square	0.99

**Table 5:** Performance Comparison of Predictive Scores.

Score	AUC (95% CI)	Sensitivity (%)	Specificity (%)	PPV (%)	NPV (%)
CRUI Score	0.87 (0.84–0.90)	82	79	76	84
Bishop Score	0.62 (0.58–0.66)	58	63	52	68
ADOLESRISK Score	0.84 (0.81–0.87)	84	76	71	87
Traditional Risk Assessment	0.68 (0.64–0.72)	65	69	58	75

**Table 6:** Performance of Predictive Scores in Training and Validation Cohorts.

Score	Cohort	N	AUC (95% CI)	Sensitivity (%)	Specificity (%)	PPV (%)	NPV (%)	Accuracy (%)	*p*-Value
	Training	925	0.88 (0.84–0.91)	83	80	78	85	81.4	-
	Validation	397	0.85 (0.80–0.90)	80	78	75	82	79.1	0.24
	Difference	-	−0.03	−3	−2	−3	−3	−2.3	Ns
BISHOP Score (Comparison)	Training	925	0.63 (0.59–0.67)	59	64	53	69	61.8	-
	Validation	397	0.61 (0.55–0.67)	57	62	51	67	59.7	0.58
Adolesrisk Score	Training	925	0.85 (0.82–0.88)	85	77	73	88	80.8	-
	Validation	397	0.82 (0.77–0.87)	82	74	69	85	77.6	0.31
	Difference	-	−0.03	−3	−3	−4	−3	−3.2	Ns
Traditional Risk Assessment (Comparison)	Training	925	0.69 (0.65–0.73)	66	70	59	76	68.2	-
	Validation	397	0.68 (0.62–0.74)	64	69	57	74	66.7	0.74

Ns-Not significant.

**Table 7:** Clinical Outcomes by ADOLESRISK Score Categories.

Risk Category	Score Range	n (%)	Major Complications (%)	Cesarean Rate (%)	NICU Admission (%)
**Low Risk**	0–7	487 (36.8)	8.2	12.1	5.7
**Moderate Risk**	8–14	595 (45.0)	23.4	26.7	18.3
**High Risk**	15–20	240 (18.2)	67.5	58.3	45.8

**Table 8:** Statistical Comparisons Between Favorable and Unfavorable Outcome Groups.

Outcome Variable	Favorable Outcome (N = 934)	Unfavorable Outcome (N = 388)	Statistical Test	*p*-Value
Demographic Characteristics				
Maternal Age (Years), Mean ± Sd	15.4 ± 0.8	15.1 ± 0.9	Independent *t*-test	<0.001
Age < 15 Years, N (%)	128 (13.7)	82 (21.1)	Chi-Square	<0.001
Rural Residence, N (%)	751 (80.4)	344 (88.7)	Chi-Square	<0.001
Secondary Education Completed, N (%)	289 (30.9)	75 (19.3)	Chi-Square	<0.001
Clinical Parameters				
BMI (kg/m^2^), Mean ± Sd	20.1 ± 2.2	19.1 ± 2.5	Independent *t*-test	<0.001
BMI < 18.5 Kg/m^2^, N (%)	221 (23.7)	149 (38.4)	Chi-Square	<0.001
Hemoglobin (g/dL), Mean ± Sd	11.2 ± 1.1	10.3 ± 1.3	Independent *t*-test	<0.001
Anemia (Hb < 11 g/dL), N (%)	364 (39.0)	231 (59.5)	Chi-Square	<0.001
Inadequate Prenatal Care, N (%)	256 (27.4)	196 (50.5)	Chi-Square	<0.001
Gestational Age at Delivery (weeks), Mean ± Sd	38.7 ± 1.8	36.8 ± 3.2	Independent *t*-test	<0.001
Ultrasound Parameters				
Cervical Length (Mm), Mean ± Sd	29.8 ± 6.2	25.1 ± 7.1	Independent *t*-test	<0.001
Posterior Cervical Angle (Degrees), Mean ± Sd	95.2 ± 11.8	104.7 ± 12.9	Independent *t*-test	<0.001
Bishop Score, Mean ± Sd	5.8 ± 2.1	4.2 ± 2.3	Independent *t*-test	<0.001
Efw < 10th Percentile, N (%)	78 (8.3)	78 (20.1)	Chi-Square	<0.001
Mode Of Delivery				
Vaginal Delivery, N (%)	807 (86.4)	169 (43.6)	Chi-Square	<0.001
Cesarean Delivery, N (%)	127 (13.6)	219 (56.4)	Chi-Square	<0.001
Spontaneous Labor, N (%)	712 (76.2)	182 (46.9)	Chi-Square	<0.001
Labor Induction, N (%)	153 (16.4)	128 (33.0)	Chi-Square	<0.001
Pregnancy Complications				
Gestational Hypertension, N (%)	98 (10.5)	67 (17.3)	Chi-Square	<0.001
Severe Preeclampsia, N (%)	41 (4.4)	92 (23.7)	Chi-Square	<0.001
Eclampsia, N (%)	3 (0.3)	15 (3.9)	Chi-Square	<0.001
Preterm Birth (<37 Weeks), N (%)	89 (9.5)	109 (28.1)	Chi-Square	<0.001
Prom, N (%)	156 (16.7)	98 (25.3)	Chi-Square	<0.001
Oligohydramnios, N (%)	78 (8.3)	64 (16.5)	Chi-Square	<0.001
Fetal Growth Restriction, N (%)	67 (7.2)	71 (18.3)	Chi-Square	<0.001
Delivery Complications				
Postpartum Hemorrhage (>500 mL), N (%)	45 (4.8)	42 (10.8)	Chi-Square	<0.001
Perineal Trauma (3rd/4th Degree), N (%)	34 (3.6)	28 (7.2)	Chi-Square	0.004
Retained Placenta, N (%)	23 (2.5)	19 (4.9)	Chi-Square	0.021
Neonatal Outcomes				
Birth Weight (G), Mean ± Sd	3087 ± 456	2721 ± 589	Independent *t*-test	<0.001
Low Birth Weight (<2500 g), N (%)	134 (14.3)	109 (28.1)	Chi-Square	<0.001
Nicu Admission, N (%)	112 (12.0)	166 (42.8)	Chi-Square	<0.001
Nicu Stay > 24 h, N (%)	89 (9.5)	134 (34.5)	Chi-Square	<0.001
Respiratory Distress, N (%)	67 (7.2)	89 (22.9)	Chi-Square	<0.001
Neonatal Sepsis, N (%)	12 (1.3)	23 (5.9)	Chi-Square	<0.001
Hypoglycemia, N (%)	78 (8.3)	67 (17.3)	Chi-Square	<0.001
Apgar Score				
Apgar 1 min, Mean ± Sd	8.1 ± 1.2	7.2 ± 1.6	Mann–Whitney U	<0.001
Apgar 5 min, Mean ± Sd	9.1 ± 0.7	8.5 ± 1.1	Mann–Whitney U	<0.001
Apgar < 7 At 5 min, N (%)	23 (2.5)	45 (11.6)	Chi-Square	<0.001
Perinatal Mortality				
Stillbirth, N (%)	2 (0.2)	4 (1.0)	Fisher Exact	0.048
Early Neonatal Death, N (%)	1 (0.1)	7 (1.8)	Fisher Exact	<0.001
Total Perinatal Mortality, N (%)	3 (0.3)	11 (2.8)	Fisher Exact	<0.001

**Table 9:** Multivariate Analysis: Independent Risk Factors for Major Obstetrical Complications.

Risk Factor	Odds Ratio	95% CI	*p*-Value	Score Points
Age < 15 years	2.34	1.78–3.08	<0.001	3
BMI < 18.5 kg/m^2^	1.89	1.45–2.47	<0.001	2
No secondary education	1.67	1.32–2.11	<0.001	2
Inadequate prenatal care	2.78	2.18–3.54	<0.001	4
Hemoglobin < 10 g/dL	2.12	1.68–2.68	<0.001	3
Gestational age < 37 weeks	3.45	2.67–4.46	<0.001	5
EFW < 10th percentile	2.89	2.21–3.78	<0.001	4

## Data Availability

The data that support the findings of this study are available from the corresponding author upon reasonable request but are not publicly available due to ethical and privacy considerations.

## Abbreviations

The following abbreviations are used in this manuscript:

**CRUI**	Cervical Ripening Ultrasound Index
**CL**	Cervical Length
**PCA**	Posterior Cervical Angle
**CES**	Cervical Echogenicity Score
**PF**	Presence of Funneling
**ROC**	Receiver Operating Characteristic
**BMI**	Body Mass Index
**AUC**	Area Under the Curve
**NICU**	Neonatal Intensive Care Unit
**EFW**	Estimated Fetal Weight
**CI**	Confidence Interval
**OR**	Odds Ratio
**HPA**	Hypothalamic–Pituitary–Adrenal (axis)
**PlGF**	Placental Growth Factor
**sFlt-1**	Soluble fms-like tyrosine kinase-1
